# The Impact of IT-Based Healthcare Communication on Mammography Screening Utilization among Women in the United States: National Health Interview Survey (2011–2018)

**DOI:** 10.3390/ijerph191912737

**Published:** 2022-10-05

**Authors:** Noof Alabdullatif, Alejandro Arrieta, Lucie Dlugasch, Nan Hu

**Affiliations:** 1 Department of Health Policy and Management, FIU Robert Stempel College of Public Health and Social Work, Miami, FL 33199, USA; 2 Department of Graduate Nursing, Nicole Wertheim College of Nursing and Health Sciences, Miami, FL 33199, USA; 3 Department of Biostatistics, FIU Robert Stempel College of Public Health and Social Work, Miami, FL 33199, USA

**Keywords:** breast cancer screening, mammography utilization, healthcare communication, information technology

## Abstract

Effective patient–provider communication improves mammography utilization. Using information technology (IT) promotes health outcomes. However, there are disparities in access to IT that could contribute to disparities in mammography utilization. This study aims to assess the association between IT-based health care communication and mammography utilization and to evaluate if this effect is modified by race/ethnicity and age. To this end, this study was conducted using the National Health Interview Survey from 2011 to 2018. A total of 94,290 women aged 40 years and older were included. Multiple logistic regression models were used, and odds ratios were reported. The study found that all IT-based healthcare communication strategies were significantly associated with mammography utilization in all years from 2011 to 2018. In 2018, women who looked up health information on the internet, scheduled a medical appointment on the internet, and communicated with providers by email had a significantly higher chance to use mammography (*p* ≤ 0.005 for all strategies across all years). White women and women aged 50 years and older benefited the most from IT-based healthcare communication. In conclusion, facilitating access to IT may help increase mammography utilization, which may contribute to eliminating disparities in breast cancer mortality.

## 1. Introduction

Breast cancer is the most common cancer and the second leading cause of cancer death among women in the United States (U.S.). Mammography is the most effective screening tool used for the early detection of breast cancer and has been shown to reduce breast cancer mortality [1]. However, the mammography screening utilization rate in the U.S. (72.8%) was still lower than the Healthy People 2020 (HP2020) national target in the most recently reported data in 2018 (81.1%) [2]. The American Cancer Society (ACS) [3] reported important disparities in the rate of mammography with non-Hispanic Blacks having the highest rate, followed by non-Hispanic Whites and Hispanics having the lowest rates, followed by non-Hispanic Asian Americans. Age, along with race and ethnicity, was another determining factor influencing screening rates. The highest screening rate was among women aged 55–74 years, and the lowest was among those aged 75 years and above. Hence, it is important to investigate the factors behind these existing disparities, which could contribute to the disparity in breast cancer mortality [3].

Effective patient–provider communication is essential to build a strong and trusting relationship that enhances health outcomes and improves patient satisfaction [4]. According to Peterson et al. [5], besides the provider’s screening recommendation, the quality of the patient–provider communication improves mammography screening utilization and encourages women’s adherence to screening guidelines. Meguerditchian et al. [6] suggested that women who saw physicians who had better communication skills were more likely to use mammography screening. Roman et al. [7] and Kindratt et al. [8] both reported racial and ethnic disparity in the impact of patient–provider communication on mammography screening. For instance, patient–provider communication was reported to not be significantly associated with mammography screening utilization among Black women [7]. In addition, an analysis stratified by age found that patient–provider communication regarding mammography screening was associated with better mammography screening utilization only among women aged 40 to 49 years [9]. Thus, active research is necessary to further understand the relationship between patient–provider communication and mammography screening utilization, better examine the related disparities in mammography screening utilization, and consider communication as an opportunity to increase mammography screening utilization equitably in the U.S.

Using IT-based healthcare communication strategies may promote population health outcomes and enhance health equity as reported in the Healthy People 2030 (HP2030) goals [10]. One of the HP2030 objectives is to “increase the proportion of adults who use IT to track health care data or communicate with providers”. This objective is defined as using a computer, smartphone, or other electronic means to do at least one of the followings; looked for health or medical information, used e-mail or the internet to communicate with a doctor or a doctor’s office, made appointments with a health care provider, and looked up test results in the past 12 months [10].

Online communication strategies are expected to be an essential part of the healthcare delivery system [11]. A study by Tan and Goonawardene [12] found that online communication with healthcare providers through the internet and/or health IT would promote the relationship between patients and providers and encourage both parties to make better-informed decisions regarding patients’ health. Liu et al. [13] found that communicating with healthcare providers using e-mail, social media, mobile apps, and health-related websites improved quality of life (QOL), including physical, mental, and social wellbeing. Additionally, Lee [14] and Zhang et al. [15] claimed that health IT could be used as a strategy to address health disparities.

Evidence-based interventions that rely on health IT have been shown to have a positive impact on mammography screening utilization, and women’s screening behavior. For example, Elkin et al. [16] and Eden et al. [17] both recommended that web-based decision aids could reduce decisional conflict about mammography screening and assist women in having informed and personalized decisions regarding screening. Another intervention is the electronic reminder system, which was found to be an effective strategy to increase women’s adherence to mammography screening guidelines and scheduled mammography appointments [18,19].

Despite the increase in health IT use, there are still disparities in patients’ access, which could potentially limit the utilization of preventive services among minority groups and underserved populations [10]. These variations could shape the differences in women’s mammography screening behaviors and contribute to the disparities in breast cancer mortality. Therefore, ensuring equitable access to those technologies and resources is crucial to supporting underserved populations.

Although previous research evaluated the impact of IT-based interventions on mammography screening utilization, there is still a gap in understanding how mammography screening utilization is associated with the routine use of IT to communicate with healthcare providers. In this study, IT-based healthcare communication is specified as looking up health information on the internet, scheduling medical appointments on the internet, or communicating with healthcare providers via email. This study used a nationally representative sample to evaluate how IT-based communication with healthcare providers is associated with mammography screening utilization. It also investigated whether race/ethnicity and age modify this relationship. Specifically, three hypotheses were examined: first, whether IT-based healthcare communication improves mammography screening utilization among women aged 40 years and older in the U.S; second, whether the relationship between IT-based healthcare communication and mammography screening utilization is modified by race/ethnicity; last, if the relationship between IT-based healthcare communication and mammography screening utilization is modified by age. In Section 2, we provide readers with information about our data source, define our study sample, specify the inclusion and exclusion criteria, and illustrate our study design and analytical methods. In this section, we also describe the study measures, including the study outcome, exposures, and confounding variables. In Section 3, we summarize the characteristics of our study cohort and report our results in regard to the association between mammography screening utilization and IT-based healthcare communication strategies, as well as the effect modification by race/ethnicity and age. In Section 4, we discuss our study findings, address the study limitations, and give future directions. In Section 5, we conclude our study by highlighting our key results and providing our recommendations to healthcare providers and policymakers.

## 2. Materials and Methods

### 2.1. Study Design and Data Resource

This study is a cross-sectional study using multiple years’ data (year 2011 to 2018). In this research, the annual cross-sectional National Health Interview Survey (NHIS) was used. NHIS is conducted by the National Center for Health Statistics (NCHS), a part of the U.S. Centers for Disease Control and Prevention (USCDC). NHIS is considered one of the principal data sources of health information in the U.S. The NHIS sample follows a multistage area probability design that involves stratification, clustering, and oversampling of some subpopulations such as Blacks, Hispanics, and Asians. Family, Sample Adult, Person, and Sample Adult Cancer data files were used in the analysis and available for public use. More details about NHIS and the data files could be found on the CDC’s NIHS website [20]. The study was conducted in accordance with the Declaration of Helsinki, and approved by the Institutional Review Board of Florida International University (protocol code: IRB-22-0299, date of approval: 24 June 2022).

### 2.2. Study Population and Sample Data

The NHIS publicly released data files include 778,493 sample adults from 2011 to 2018. The targeted population includes only women aged 40 years and older (n = 94,290) to assess the association between IT-based healthcare communication and mammography screening utilization and to investigate the effect modification by race/ethnicity and age. The analysis is limited to this age range because it is the age that the general population of women are recommended to start mammography screening according to the breast cancer screening guidelines published by the Centers for Disease Control and Prevention (CDC) [21].

### 2.3. Variables

#### 2.3.1. Outcomes

The outcome of this study is the self-reported mammography screening utilization in the past 12 months (Y). All women who were selected to complete the “Sample Adult” questionnaire and aged 30 years and older were asked the following question: “Have you had a mammogram during the past 12 months?” This binary question (yes or no) is available from 2011 to 2017. However, for the year 2018, women were asked about the most recent mammogram (a year ago or less, more than 1 year but not more than 2 years, more than 2 years but not more than 3 years, more than 3 years but not more than 5 years, or over 5 years ago). To create a variable that aligns with the outcome (Figures S1 and S2), this variable was dichotomized in which only women who had their most recent mammogram within a year ago or less were coded as “yes”.

#### 2.3.2. Exposures

The exposure is the self-reported IT-based health care communication in the past 12 months. The exposure variables are available in the “Sample Adult” questionnaire and are indicated by three questions (Q_1_, Q_2_, and Q_3_). Each question asked for the usage of one IT strategy to communicate with women’s healthcare providers during the past 12 months:

Q_1_: “Have you ever used computers to look up health information on Internet?”

Q_2_: “Have you ever used computers to schedule a medical appointment on Internet?”

Q_3_: “Have you ever used computers to communicate with a healthcare provider by email?”

All IT-based healthcare communication questions (Q_1_–Q_3_) were binary variables (yes or no). Those questions were used in the analysis in three different ways to assess the association between IT-based healthcare communication and mammography screening utilization. First, each question (Q_1_–Q_3_) was analyzed as an independent strategy that represents IT-based healthcare communication. In addition, “Composite” exposure to IT-based healthcare communication was defined as using at least one of the four communication strategies Q_4_ (“yes” if any of the answers from Q_1_–Q_3_ is “yes”).

Finally, the number of strategies used (on a 0–3 scale) were defined as another exposure, where zero indicates no communication strategies were used and three reflects that all of the communication strategies (Q_1_–Q_3_) were used by a study subject (nstg). This is an ordinal categorical variable and the categorical 0 was used as the reference level in the analysis.

#### 2.3.3. Confounding Variables

Many confounding variables were included in the models the following domains: demographic, socioeconomic, and women’s health and behavior factors. Patients’ demographic features includes age groups (“age” in (Equation (1)) in Section 2.4.) is categorized into two groups (40–49 years and 50+ years)), race/ethnicity (“race” in Equation (1)) which includes four groups (Hispanic, non-Hispanic White, non-Hispanic Black, and non-Hispanic other including all other race groups such as Asians), marital status (“ms” in Equation (1)) which is a binary variable (currently married and otherwise); regions (“reg” in Equation (1)) that is categorized into four groups (Northeast, Midwest, South, and West), and study subjects’ insurance coverage (“ins” in Equation (1)) which includes five categories (private, public, military, other, and uninsured). Socioeconomic status variables includes the ratio of family income to the poverty threshold (“pov” in Equation (1)) which is categorized into four groups (<100%; 100%–199%; 200%–399%; and ≥ 400%), education (“educ” in Equation (1)) which is categorized into five groups (less than high school; high school; some college or associate degree; bachelor’s degree; and graduate degree), and whether (yes or no) working for pay in the past 12 months (“wrk” in Equation (1)). The variables related to women’s health and behaviors includes physical health status (“hlths” in Equation (1)) which is categorized into 5 groups (excellent, very good, good, fair, and poor), whether (yes or no) having a place usually go when sick (“usc” in Equation (1)), whether (yes or no) seen or talked to a general doctor in the past 12 months (“dr” in Equation (1)), and whether (yes or no) seen or talked to OB/GYN in the past 12 months (“obg” in Equation (1)). It was adjusted for the year of the survey as a categorical variable for every year from 2011 to 2018 only in the analysis of all years combined. It was also controlled for mammography recommendation “*rec*” as a binary variable (yes or no) in year 2015 due to the availability of this variable. All confounding variables were self-reported.

### 2.4. Statistical Analysis

Data are summarized as frequency (N) and percentage (%) for all variables. Mammography screening utilization was assessed using multiple logistic regression models [22]. Odds ratios were reported to measure the ratio of odds of utilizing mammography screening among women communicating with healthcare providers using IT versus among those who did not. The equation for the multivariable logistic model that was used in this study can be written as:

(1) $$\mathrm{Logit}[P\left(i_{Y}=1\right)]=\log\;\frac{P\left(Y_{i}=1\right)}{1-P\left(Y_{i}=1\right)}=\beta_{0}+\beta 1_{\;}Q_{\mathrm{ij}}+\beta_{2}\;\mathrm{age}+\beta_{3}\;{\mathrm{race}}_{i}+\beta_{4}\;{\mathrm{ms}}_{i}+\beta_{5}\;{\mathrm{reg}}_{i}+\beta_{6}\;{\mathrm{ins}}_{i}+\;\beta_{7}\;{\mathrm{educ}}_{i}\;+\;\beta_{8}\;{\mathrm{wrk}}_{i}\;+\;\beta_{9}\;{\mathrm{dr}}_{i}\;+\;\beta_{10}\;{\mathrm{obg}}_{i}\;+\;\beta_{11}\;{\mathrm{pov}}_{i}\;+\;\beta_{12}\;{\mathrm{usc}}_{i}\;+\;\beta_{13}\;{\mathrm{hlths}}_{i}$$

where *I* (*I* = 1, …, n) is the index of study participants in the dataset and n = 94,290. Y*_i_* is the dummy variable indicating the mammography screening utilization in the past 12 months and *Q_ij_* (*j* = 1, 2, 3, and 4) indicates the three strategies representing the IT-based healthcare communication and the Composite IT-based healthcare communication. Among the independent variables in model (1), *Q_ij_* (for each *j* in 1, 2, 3 and 4) is the exposure of subject *i*, and all other variables are confounding variables defined in Section 2.3.3. Exp (*β*_0_) is the odds of utilizing mammography screening (Mamuse = 1) for women who did not communicate with healthcare providers using IT (*Q_ij_* = 0) when other covariates = 0 and other factors at the reference level. *β*_1_ is the log of odds ratio comparing women who communicated with healthcare providers using IT versus those who did not when all other covariates and/or factors are held constant.

Trend analysis was used to investigate the probability of using mammography screening based on the number of IT strategies (nstg). To assess if there is a difference among race/ethnicity in the relationship between IT-based healthcare communication and mammography screening utilization, race/ethnicity (Hispanic; NH White; NH Black; and NH other) was used as an effect modifier in the model with defining “NH White” as the reference group (baseline). Three dummy variables (0: No and 1: Yes) for “Hispanic”, “NH Black”, and “NH other” were created to represent the four categories of the race variable. Three variables for each question (Q_1_–Q_4_) that were equal to the product of the question variable with the race/ethnicity dummy variables were generated. For example, for*Q_ij_*, *Q_ij_* × Hispanic, *Q_ij_* × NH Black, and *Q_ij_* NH other were created where (*i* = 1, …, n) and Q*_ij_* (e.g., *Q_i_*_2_). Those product variables were included in the model.

To assess the effect modification by age in the relationship between IT-based healthcare communication and mammography screening utilization, age “40–49 years/50+ years” was used as an effect modifier in the model with defining “40–49 years” as the reference group (baseline). The method used to create interaction terms is the same as for the exposure and race/ethnicity interaction.

Complete case analysis was used to remove the small proportion (4.37%) of missing observations from the analysis. All analyses were conducted using Stata (Stata Corp., College Station, TX, USA) version 16. Results with *p*-value < 0.05 are considered statistically significant. All reporting follows the Strengthening the Reporting of Observational Studies in Epidemiology Statement checklist (STROBE) [23].

## 3. Results

### 3.1. Characteristics of Study Participants

Descriptive statistics of women aged 40 years and above (n = 94,290) are shown in Table 1. With this age constraint, about 23% were between 40 and 49 years, and 77% were 50 years and older. Most women were non-Hispanic White women (67.71%) and privately insured (59.73). The majority had a usual source of care (93.49%) and had seen or talked to a general doctor in the past 12 months (78.93%). About fifty-two percent of the women (52.39%) used mammograms as a screening tool in the past 12 months (Table S1). Less than half of them (46.29%) reported using at least one IT-based healthcare communication strategy; forty-five percent looked up health information on the internet, seven percent scheduled medical appointments on the internet, and ten percent communicated with a healthcare provider by email (Table S2). As for the number of IT strategies used, fifty-four percent reported not using any of the IT-based healthcare communication strategies and only four percent reported using the three strategies (Table S2).

### 3.2. Associations between IT-Based Healthcare Communication Strategies and Mammography Screening Utilization

Based on the results from the univariable logistic regressions, the associations (unadjusted) between the IT-based healthcare communication strategies and mammography screening utilization were statistically significant during the entire study period (*p* < 0.001 in all years, Table S3). The adjusted odds ratios (oRs) and their 95% confidence intervals (cIs) are presented in Table 2. All IT-based healthcare communication strategies were statistically significantly associated with mammography screening utilization during the study period. The results indicated that women who looked up health information on the internet (Q_1_) were more likely to use mammography screening (*p* ≤ 0.010 in all years). There was a 36% increase in the odds of using mammography screening among women looking up health information on the internet in 2018 (OR: 1.36, 95% CI: 1.23–1.50). “Scheduling medical appointment on the internet” (Q_2_) was significantly associated with mammography screening utilization (*p* ≤ 0.020 across all years). In 2018, scheduling medical appointments on the internet increased the odds of using mammography screening by 21% (OR: 1.21, 95% CI: 1.06–1.39). “Communicating with healthcare providers by email” (Q_3_) also showed a significant increase in the odds of using mammography screening (*p* < 0.031 across all years). The increase in the odds was 34% in 2018 (OR: 1.34, 95% CI:1.18–1.52). Finally, significant associations were also observed between using at least one of the above IT-based communication strategies (Q_4_) and mammography screening utilization (*p* < 0.001 across all years). The odds of utilization in 2018 increased by 43% among women who use at least one of the strategies (OR: 1.43, 95% CI: 1.28–1.59).

### 3.3. Associations between IT-Based Healthcare Communication Strategies and Mammography Screening Utilization after Adjusting for Mammography Recommendation

After adjusting for mammography recommendation using only 2015 screening outcome data, Q_1_, Q_2_, Q_3_, and Q_4,_ showed a significant association with mammography screening utilization (*p* ≤ 0.049 for all the above strategies) (Table 3).

### 3.4. The Predicted Probability of Mammography Screening Utilization by the Number of IT-Based Healthcare Communication Strategies

**Figure 1:**
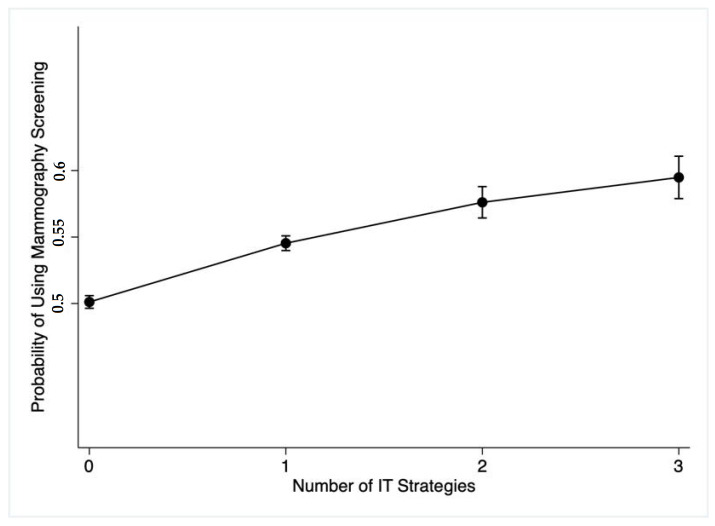
Predicted Probability of Mammography Screening versus. The number of IT Strategies.

The analysis showed a statistically significant linear trend in the probability of using mammography screening and the number of IT-based healthcare communication strategies (nstg) (*p* < 0.001, Table S4). Figure 1. Showed that the more IT-based strategies used to communicate with healthcare providers the higher the probability of using mammography screening.

### 3.5. Effect Modification by Race/Ethnicity on the Association between IT-Based Healthcare Communication and Mammography Utilization

The difference among race/ethnicity in the relationship between IT-based healthcare communication strategies and mammography screening utilization was evaluated (Table 4). For Q_1_, Q_3_, and Q_4_, the relationship between IT-based healthcare communication and mammography screening utilization was significantly different between Hispanic women and NH Whites (*p* ≤ 0.007 for Q_1_, Q_3_, and Q_4_). For all these strategies, Hispanic women benefited less than NH White women (OR equals to 0.76, 0.72, and 0.75, respectively). For Q_2_, no significant difference was found between Hispanic women and NH White. When comparing NH Black women to NH Whites, a significant difference was only observed for Q_1_ and Q_4_ in which NH Black women benefited less (OR equals to 0.84 and 0.85, respectively, *p* < 0.001 for both). There is no significant difference in the effect of all IT-based healthcare communication strategies (Q_1_–Q_4_) on mammography screening utilization between women of NH White and other NH races.

### 3.6. Effect Modification by Age on the Association between IT-Based Healthcare Communication and Mammography Utilization

Finally, it was examined if age modifies the relationship between IT-based healthcare communication strategies and mammography screening utilization. The relationship between all IT-based healthcare communication strategies or the composite (Q_1_–Q_4_) and mammography screening utilization was significantly different between women aged 50+ years and those aged 40–49 years (*p* < 0.001 for all the above strategies, Table 5). Compared to women aged 40–49 years, older women aged 50 years and above (50+) had a greater benefit from all strategies (oRs are 1.85, 1.35, 1.34 and 1.67, for Q_1_–Q_4_, respectively).

## 4. Discussion

The study found that all IT-based healthcare communication strategies separately and compositely were statistically significantly associated with mammography utilization across all years from 2011 to 2018. NH White women and those aged 50 years and above benefited greater from the IT-based healthcare communication than younger women and those from other races/ethnicities such as Hispanics and NH Blacks. This is among the first studies to evaluate the association between IT-based healthcare communication and mammography utilization in the U.S. This study is the first to describe how this association is modified by women’s race/ethnicity and age. This study provides externally valid results using a large nationally representative sample that is intended to represent U.S. populations.

The analyses revealed that looking up health information on the internet is associated with using mammography screening (*p* ≤ 0.009 across all years). In 2018, the odds of mammography utilization among women who looked up health information on the internet are 1.36 times the odds of utilization among those who did not (*p* < 0.001). Women who are using the internet to search for health information could be more engaged and have better health literacy, and thus more probable to use mammography screening [24]. The results support what was demonstrated by Tan and Goonawardene [12] that seeking health information on the internet promotes patient–physician communication and encourages patients to make better and informed decisions about their health. Improving internet access and enhancing women’s education to search health information on the internet may contribute to improving mammography screening utilization.

Significant associations were also found between scheduling a medical appointment using the internet and mammography screening utilization (*p* ≤ 0.020 across all years). In 2011, the odds of utilizing mammography among women who scheduled medical appointments using the internet were 1.43 times the odds of the utilization among those who did not (*p* < 0.004). Facilitating scheduling appointments through the internet, especially for preventive services such as mammography screening is expected to encourage women’s adherence and improve their utilization. We also recommend considering the challenges associated with online self-scheduling such as patient awareness, communication preference, and distrust of the internet [25].

The study also showed a significant association between communication with healthcare providers via email and mammography utilization (*p* < 0.031 across all years). For example, the increase in the odds of utilization was 61% among women who communicated with healthcare providers via email compared to those who did not in 2017 (*p* < 0.001). Our results are consistent with a previous study that used the same database (NHIS data from 2011 to 2015) and concluded that women who used email to communicate with healthcare providers are more likely to use mammography screening [26]. However, another study that used another national survey (Health Information National Trends Survey from 2011 to 2014) found no significant associations between email communication and breast cancer screening [27]. Given the conflicting results, more research is needed to investigate this association.

Using at least one of the IT-based healthcare communication strategies was highly associated with mammography screening utilization (*p* ≤ 0.001 across all years). In 2018, the odds of mammography utilization among women who used at least one of the IT-based healthcare communication strategies were 1.44 times the utilization among those who did not (*p* < 0.001). All IT-based healthcare communication strategies remained statistically significantly associated with mammography utilization after accounting for doctor recommendations for mammography in the analysis of the year 2015.

To the best of our knowledge, this is the first study to analyze the trend of the probability of using mammography screening by the number of IT-based healthcare communication strategies. Using more IT-based healthcare communication strategies is expected to increase the probability of using mammography screening. For instance, using three IT strategies was better than using two strategies in terms of mammography utilization. Similarly, Sims et al. [28] found that using more technologies is positively associated with better health outcomes and higher life satisfaction among older adults.

Effect modification by race aims to investigate whether the effect of IT-based healthcare communication on mammography utilization differs among racial/ethnic groups. Hispanic women benefited less than NH Whites from looking up health information on the internet, communicating with healthcare providers via email, and using at least one of the IT-based healthcare communication strategies. Additionally, NH Black women benefited less than NH Whites from looking up health information on the internet and using at least one of the IT-based healthcare communication strategies. Unlike our results, Lee [14] found that the positive impact of health IT investment was greater for non-Whites than for Whites. In this sample, Hispanic and NH Black women aged 40 years and above were adverse in many factors such as socioeconomic, insurance coverage, and health status. Disparities in those factors may lead to significant variations in mammography screening utilization among different ethnic groups. For instance, Among NH Whites, about 10% were from the lowest family income ratio to the poverty level (<100%) and about 42% were from the highest (≥400, Table S5). Among NH Blacks, 28% were from the lowest income ratio to the poverty level (<100%) and about 18% were from the highest (≥400, Table S5). Similarly, around 12% of NH White women had less than a high school degree and about 30% had a bachelor’s or graduate degree (Table S6). Among Hispanic women, around 44% had less than a high school degree and 14% had a Bachelor’s or graduate degree (Table S6). Insurance disparity was also found among different ethnic groups. Among the uninsured women in this sample, 30% were Hispanic. Most privately insured women were NH White (75.5%) followed by NH Black (11%), and Hispanic (8%, Table S7). Thus, more research is needed to investigate these ethnic disparities and to understand how using IT affects the health behaviors and health outcomes of racial/ethnic minorities and underserved populations.

We also conducted a mediation analysis to investigate whether income mediates the relationship between IT-based healthcare communication and mammography screening utilization [29]. We found that income mediates the relationship between looking-up health information on the internet (Q_1)_ and mammography screening utilization. (Tables S9–S12)

Effect modification by age aims to examine whether the effect of IT-based healthcare communication on mammography utilization differs between different age groups. A highly significant difference was found. Compared to women aged 40–49 years, those aged 30–39 years benefited less whereas older women aged 50+ benefited greater from looking up health information on the internet, scheduling medical appointments on the internet, communicating with healthcare providers via email, and using at least one of the IT-based healthcare communication strategies. Interventions to improve mammography utilization should be tailored for racial/ethnic groups and age groups who benefited less from using health IT.

### Study Limitations

This study has some limitations. First, we could not interpret our results from year 2011 to 2018 as a pattern because the NHIS design is not longitudinal and it changed from one year to another, and thus differences in the results between years could be due to sampling error. Second, this is a cross-sectional study to build an association relationship, and causality could not be established based on our results. Third, we used secondary data, which limited our analysis of what is available. For instance, health IT questions in the NHIS were started with “Did you use computers to do one of the following”, and that could have affected women’s answers if they were using other devices such as tablets or smartphones. Fourth, our results are built on self-reported data and are thus prone to social desirability bias. Last, although we discussed our results based on each IT-based healthcare communication strategy, these strategies are not mutually exclusive. For example, women can schedule a medical appointment using email.

More research is needed to further understand the relationship between IT usage and mammography screening utilization. Future studies should also investigate the effect of using Telehealth to communicate with healthcare providers on mammography screening utilization. Intervention studies (e.g., short videos or educational sessions) may help to target underserved populations and less educated communities to improve their IT-based healthcare communication strategies.

## 5. Conclusions

The results suggest that using IT-based healthcare communication strategies is statistically significantly associated with mammography screening utilization. NH White women and older women benefited greater from IT-based healthcare communication. Improving equitable access to IT and educating women aged 40 years and older on how to use IT-based strategies may help to enhance patient–provider communication and hence improve mammography screening utilization and contribute to eliminate health disparity. Decision-makers should consider implementing IT interventions that are tailored for age or racial/ethnic groups who benefited less from IT usage.

## Figures and Tables

**Table 1:** Descriptive statistics of the demographics and socioeconomic characteristics among women aged 40+, 2011–2018 (n = 118,034).

Variable		Frequency (N)	Percentage (%)
Age group	40–49	21,645	22.96
	50+	72,645	77.04
Race/ethnicity	Hispanic	11,548	12.25
	NH White ^a^	63,843	67.71
	NH Black ^b^	13,287	14.09
	NH Other ^c^	5612	5.95
Marital Status	Currently married	40,382	42.83
	Otherwise	53,908	57.17
Region	Northeast	16,527	17.53
	Midwest	20,246	21.47
	South	34,456	36.54
	West	23,061	24.46
Insurance Coverage	Private	56,171	59.73
	Public	25,374	26.98
	Military	3350	3.56
	Other	1325	1.41
	Uninsured	7814	8.31
Education	Less than high school	16,769	17.88
	High school	22,561	24.05
	Some college	28,120	29.98
	Bachelor’s degree	15,940	16.99
	Graduate degree	10,408	11.10
Ratio of Family Income to the Poverty Threshold	<100%	12,863	15.23
	100–199%	17,511	20.73
	200–399%	23,969	28.38
	≥400%	30,113	35.66
Work Status	Yes	46,519	49.36
	No	47,718	50.64
Survey Year	Year 2011	11,574	12.27
	Year 2012	12,306	13.05
	Year 2013	12,364	13.11
	Year 2014	13,436	14.25
	Year 2015	12,483	13.24
	Year 2016	12,253	13.00
	Year 2017	10,099	10.71
	Year 2018	9775	10.37

^a^ NH White: Non-Hispanic White. ^b^ NH Black: Non-Hispanic Black. ^c^ NH other: Non-Hispanic other.

**Table 2:** Odds ratio and 95% CI based on multiple logistic regression, 2011–2018. Adjusted model 1 ^a^.

Mammography Utilization by Time Period	Odds Ratio	S.E.	*p*	95% Conf. Interval	
All years 2011–2018 (n = 94,290) ^b^					
Q_1_ ^c^	1.22	0.02	<0.001	1.18	1.27
Q_2_ ^d^	1.23	0.04	<0.001	1.16	1.30
Q_3_ ^e^	1.33	0.03	<0.001	1.26	1.40
Q_4_ ^f^	1.27	0.02	<0.001	1.23	1.32
Year 2011 (n = 11,574)					
Q_1_ ^c^	1.20	0.06	0.001	1.08	1.34
Q_2_ ^d^	1.43	0.18	0.004	1.12	1.83
Q_3_ ^e^	1.26	0.13	0.029	1.02	1.56
Q_4_ ^f^	1.24	0.07	<0.001	1.12	1.38
Year 2012 (n = 12,306)					
Q_1_ ^c^	1.14	0.06	0.010	1.03	1.26
Q_2_ ^d^	1.40	0.17	0.005	1.10	1.77
Q_3_ ^e^	1.25	0.13	0.031	1.02	1.53
Q_4_ ^f^	1.16	0.06	<0.001	1.05	1.29
Year 2013 (n = 12,364)					
Q_1_ ^c^	1.15	0.06	0.004	1.05	1.27
Q_2_^d^	1.24	0.12	0.020	1.03	1.50
Q_3_ ^e^	1.32	0.11	0.001	1.12	1.56
Q_4_ ^f^	1.16	0.06	0.001	1.05	1.28
Year 2014 (n = 13,436)					
Q_1_ ^c^	1.29	0.06	<0.001	1.18	1.41
Q_2_ ^d^	1.35	0.13	0.002	1.12	1.63
Q_3_ ^e^	1.41	0.12	<0.001	1.20	1.65
Q_4_ ^f^	1.33	0.06	<0.001	1.22	1.46
Year 2015 (n = 12,483)					
Q_1_ ^c^	1.23	0.06	<0.001	1.21	1.35
Q_2_ ^d^	1.32	0.10	<0.001	1.13	1.53
Q_3_ ^e^	1.25	0.09	0.002	1.09	1.43
Q_4_ ^f^	1.27	0.06	<0.001	1.16	1.40
Year 2016 (n = 12,253)					
Q_1_ ^c^	1.28	0.06	<0.001	1.17	1.41
Q_2_ ^d^	1.29	0.09	0.001	1.12	1.49
Q_3_ ^e^	1.48	0.10	<0.001	1.30	1.69
Q_4_	1.30	0.06	<0.001	1.18	1.42
Year 2017 (n = 10,099)					
Q_1_ ^c^	1.30	0.07	<0.001	1.18	1.44
Q_2_^d^	1.19	0.09	0.019	1.03	1.38
Q_3_ ^e^	1.61	0.11	<0.001	1.40	1.84
Q_4_ ^f^	1.38	0.07	<0.001	1.25	1.53
Year 2018 (n = 9775)					
Q_1_ ^c^	1.36	0.07	<0.001	1.23	1.50
Q_2_ ^d^	1.21	0.08	0.005	1.06	1.39
Q_3_ ^e^	1.34	0.09	<0.001	1.18	1.52
Q_4_ ^f^	1.43	0.08	<0.001	1.28	1.59

^a^ Adjusted model 1: adjusting for age, race/ethnicity, marital status, education, region, insurance coverage, work status, place usually go when sick, seen/talked to a general doctor, seen/talked to OB/GYN, ratio of family income to the poverty threshold, and physical health status. ^b^ All years 2011–2018: The year of the survey was included as a control variable in the analysis for all years combined. ^c^ Q_1_: Looked-up health information on the internet (“yes” versus “no”, and “no” is the reference). ^d^ Q_2_: Scheduled medical appointment on the internet (“yes” versus “no”, and “no” is the reference). ^e^ Q_3_: Communicated with healthcare provider by email (“yes” versus “no”, and “no” is the reference). ^f^ Q_4_: Composite IT-based healthcare communication. This is coded as “yes” if at least one condition in Q_1_–Q_3_ was met and coded as “no” otherwise. “no” is the reference.

**Table 3:** Multiple logistic regression of mammography utilization based on IT-based healthcare communication with and without adjusting for mammography recommendation in 2015 (n = 12,483).

Mammography Utilization	Odds Ratio	S.E.	*p*	95% Conf. Interval	
Q_1_ ^a^					
Adjusted model 1 ^b^	1.23	0.06	<0.001	1.12	1.35
Adjusted model 2 ^c^	1.15	0.06	0.006	1.04	1.27
Q_2_ ^d^					
Adjusted model 1 ^b^	1.32	0.10	<0.001	1.12	1.54
Adjusted model 2 ^c^	1.34	0.12	0.001	1.13	1.59
Q_3_ ^e^					
Adjusted model 1 ^b^	1.25	0.09	0.002	1.08	1.43
Adjusted model 2 ^c^	1.16	0.09	0.049	1.00	1.35
Q_4_ ^f^					
Adjusted model 1 ^b^	1.27	0.06	<0.001	1.16	1.40
Adjusted model 2 ^c^	1.18	0.06	0.001	1.07	1.31

^a^ Q_1_: Looked-up health information on the internet (“yes” versus “no”, and “no” is the reference). ^b^ Adjusted model 1: adjusting for age, race/ethnicity, marital status, education, region, insurance coverage, work status, place usually go when sick, seen/talked to a general doctor, seen/talked to OB/GYN, ratio of family income to the poverty threshold, and physical health status. ^c^ Adjusted model 2: adjusting for age, race/ethnicity, marital status, education, region, insurance coverage, work status, place usually go when sick, seen/talked to a general doctor, seen/talked to OB/GYN, ratio of family income to the poverty threshold, physical health status, and doctor recommendation for mammography (“yes” versus “no”, and “no” is the reference). ^d^ Q_2_: Scheduled medical appointment on the internet (“yes” versus “no”, and “no” is the reference). ^e^ Q_3_: Communicated with healthcare provider by email (“yes” versus “no”, and “no” is the reference). ^f^ Q_4_: Composite IT-based healthcare communication. This is coded as “Yes” if at least one condition in Q_1_–Q_3_ were met and coded as “no” otherwise. “no” is the reference.

**Table 4:** Multiple logistic regression of mammography utilization based on IT-based healthcare. communication; effect modified by race, Baseline: NH White ^a^ (n = 94,290). Adjusted model 3 ^b^.

Mammography Utilization	Odds Ratio	S.E.	*p*	95% Conf. Interval	
Q_1_ ^c^					
Exposure effect	1.23	0.02	<0.001	1.18	1.27
Baseline difference					
Hispanic	1.55	0.04	<0.001	1.46	1.64
NH Black ^d^	1.54	0.04	<0.001	1.46	1.63
NH other ^e^	1.05	0.04	0.186	0.97	1.14
Effect modification by race					
Hispanic vs. NH White ^a^	0.76	0.03	<0.001	0.70	0.84
NH Black ^d^ vs. NH White ^a^	0.84	0.04	<0.001	0.77	0.92
NH other ^e^ vs. NH White ^a^	0.93	0.06	0.275	0.83	1.05
Q_2_ ^f^					
Exposure effect	1.14	0.03	<0.001	1.08	1.21
Baseline difference					
Hispanic	1.38	0.03	<0.001	1.32	1.45
NH Black ^d^	1.41	0.03	<0.001	1.35	1.48
NH other ^e^	0.98	0.03	0.497	0.92	1.04
Effect modification by race					
Hispanic vs. NH White ^a^	0.86	0.08	0.106	0.73	1.03
NH Black ^d^ vs. NH White ^a^	1.02	0.08	0.768	0.87	1.20
NH other ^e^ vs. NH White ^a^	1.18	0.12	0.102	0.97	1.43
Q_3_ ^g^					
Exposure effect	1.28	0.03	<0.001	1.22	1.35
Baseline difference					
Hispanic	1.41	0.03	<0.001	1.34	1.48
NH Black ^d^	1.43	0.03	<0.001	1.36	1.49
NH Other ^e^	1.01	0.03	0.835	0.94	1.07
Effect modification by race					
Hispanic vs. NH White ^a^	0.72	0.06	<0.001	0.60	0.86
NH Black ^d^ vs. NH White ^a^	0.95	0.08	0.574	0.81	1.12
NH other ^e^ vs. NH White ^a^	0.95	0.09	0.620	0.79	1.15
Q_4_^h^					
Exposure effect	1.28	0.02	<0.001	1.23	1.33
Baseline difference					
Hispanic	1.57	0.05	<0.001	1.48	1.67
NH Black ^d^	1.55	0.04	<0.001	1.47	1.64
NH other ^e^	1.07	0.04	0.105	0.98	1.16
Effect modification by race					
Hispanic vs. NH White ^a^	0.75	0.03	<0.001	0.69	0.83
NH Black ^d^ vs. NH White ^a^	0.85	0.04	<0.001	0.78	0.93
NH other ^e^ vs. NH White ^a^	0.93	0.06	0.212	0.82	1.04

^a^ NH White: Non-Hispanic White. ^b^ Adjusted model 3: adjusting for age, race/ethnicity, marital status, education, region, insurance coverage, work status, place usually go when sick, seen/talked to a general doctor, seen/talked to OB/GYN, ratio of family income to the poverty threshold, physical health status, year of survey, Hispanic vs. non-Hispanic, NH Black vs. non-Black, and NH other vs. non-other. ^c^ Q_1_: Looked-up health information on the internet (“yes” versus “no”, and “no” is the reference). ^d^ NH Blacks: Non-Hispanic Black. ^e^ NH other: Non-Hispanic other. ^f^ Q_2_: Scheduled medical appointment on the internet (“yes” versus “no”, and “no” is the reference). ^g^ Q_3_: Communicated with healthcare provider by email (“yes” versus “no”, and “no” is the reference). ^h^ Q_4_: Composite IT-based healthcare communication. This is coded as “Yes” if at least one condition in Q_1_-Q_3_ were. met and coded as “No” otherwise.

**Table 5:** Multiple logistic regression of mammography utilization based on IT-based healthcare communication; effect modified by age, Baseline: 40–49 years (n = 94,290). Adjusted model 4 ^a^.

Mammography Utilization	Odds Ratio	S.E.	*p*	95% Conf. Interval	
Q_1_^b^					
Exposure effect	0.72	0.02	<0.001	0.69	1.76
Baseline difference					
50+	2.71	0.06	<0.001	2.58	0.76
Effect modification by age					
50+ vs. 40–49	1.85	0.05	<0.001	1.74	1.96
Q_2_ ^c^					
Exposure effect	1.06	0.03	0.016	1.01	1.12
Baseline difference					
50+	3.29	0.07	<0.001	3.16	3.42
Effect modification by age					
50+ vs. 40–49	1.35	0.02	<0.001	1.30	1.40
Q_3_ ^d^					
Exposure effect	1.14	0.03	<0.001	1.09	1.19
Baseline difference					
50+	3.31	0.07	<0.001	3.18	3.44
Effect modification by age					
50+ vs. 40–49	1.34	0.02	<0.001	1.29	1.39
Q_4_ ^e^					
Exposure effect	0.80	0.02	<0.001	0.76	0.84
Baseline difference					
50+	3.88	0.07	<0.001	2.75	3.02
Effect modification by age					
50+ vs. 40–49	1.67	0.05	<0.001	1.57	1.76

^a^ Adjusted model 4: adjusting for age, race/ethnicity, marital status, education, region, insurance coverage, work status, place usually go when sick, seen/talked to a general doctor, seen/talked to OB/GYN, ratio of family income to the poverty threshold, physical health status, year of survey, and 50+ vs. 40–49. ^b^ Q_1_: Looked-up health information on the internet (“yes” versus “no”, and “no” is the reference). ^c^ Q_2_: Scheduled medical appointment on the internet (“yes” versus “no”, and “no” is the reference). ^d^ Q_3_: Communicated with healthcare provider by email (“yes” versus “no”, and “no” is the reference). ^e^ Q_4_: Composite IT-based healthcare communication. This is coded as “Yes” if at least one condition in Q_1_-Q_3_ were met and coded as “No” otherwise.

## Data Availability

The data presented in this study are openly available at CDC’s NIHS website at “https://www.cdc.gov/nchs/nhis/data-questionnaires-documentation.htm (accessed on 28 September 2021)” [20].

## Supplementary Materials

The following supporting information can be downloaded at: https://www.mdpi.com/article/10.3390/ijerph191912737/s1, Figure S1: Confounding Variable; Figure S2: Mediator Variable; Table S1: Descriptive statistics of the health and health behavior factors among women aged 40+, 2011–2018 (n = 118,034); Table S2: Descriptive statistics of IT-based healthcare communication strategies variables among women aged 30+, 2011–2018 (n = 118,034); Table S3: Unadjusted logistic regression of mammography utilization based on IT-based healthcare communication. Unadjusted model 0 ^a^; Table S4: Linear Trend Analysis in the Probability of Using Mammography Screening based on the Number pf IT Strategies; Table S5: Percentages of the ratio of family income to the poverty threshold based on race/ethnicity for women 40+; Table S6: Percentages of the education based on race/ethnicity for women 40+; Table S7: Percentages of the ratio of family income to the poverty threshold based on race/ethnicity for women 40+; Table S8: Percentages of the health status based on race/ethnicity for women 40+; Table S9: Odds ratio and 95% CI based on multiple logistic regression for all years combined (2011–2018) showing all covariates (n = 94,290). Adjusted model 1 ^a^; Table S10: Odds ratio and 95% CI based on multiple logistic regression for all years combined (2011–2018) showing all covariates (n = 94,290). Adjusted model 1 ^a^; Table S11: Odds ratio and 95% CI based on multiple logistic regression for all years combined (2011–2018) showing all covariates (n = 94,290). Adjusted model 1 ^a^; Table S12: Odds ratio and 95% CI based on multiple logistic regression for all years combined (2011–2018) showing all covariates (n = 94,290). Adjusted model 1 ^a^.
