# Supplementary material for: The Impact of IT-Based Healthcare Communication on Mammography Screening Utilization among Women in the United States: National Health Interview Survey (2011–2018)
Source: Int J Environ Res Public Health. 2022 Oct 5;19(19):12737. doi: 10.3390/ijerph191912737 (PMC9566602; doi:10.3390/ijerph191912737)
Supplement: Supplementary file 1 [file ijerph-19-12737-s001.zip › ijerph-1873674-supplementary.pdf]

Supplemental Materials

1. *Descriptive statistics*

   a. Health and health behavior factors

Most women in our sample had a usual source of care (93.49%) and seen or talked to a general doctor in the past 12 months (78.93%). Twenty two percent of them reported excellent health status.

Table S1. Descriptive statistics of the health and health behavior factors among women aged 40+, 2011-2018 (n=118,034).

Variable	Frequency (N)	Percentage (%)
Reported Health Status		
Excellent	18,424	19.55
Very good	29,026	30.80
Good	28,452	30.19
Fair	13,702	14.54
Poor	4,630	4.91
Usual Source of Care		
Yes	87,549	93.49
No	6,095	6.51
Seen/talked to a General Doctor		
Yes	73,533	78.93
No	19,624	21.07
Seen/talked to OB/GYN		
Yes	27,553	29.60
No	65,539	70.40
Doctor Recommendation for Mammography [a]		
Yes	14,537	61.94
No	8,931	38.06
Mammography Utilization (past 12 months)		
Yes	47,791	52.39
No	43,427	47.61

[a] Doctor Recommendation for Mammography: included only in 2015 analysis, (n=14,315).

b. IT-based healthcare strategies

About half of the women (49.61%) of the women reported using at least one IT-based healthcare communication strategy; forty-nine percent looked up health information on the internet, eight percent scheduled medical appointments on Internet, and ten percent communicated with a healthcare provider by email. As for the number of IT strategies used, fifty percent reported not to use any of the IT-based healthcare communication strategies and only four percent reported using the three strategies.

Table S2. Descriptive statistics of IT-based healthcare communication strategies variables among women aged 30+, 2011-2018 (n= 118,034).

Variable	Frequency (n)	Percent (%)
**IT-based Healthcare Communication Strategies**		
**Q_1 [a]**		
Yes	42,156	45.38
No	50,741	54.62
**Q_2 [b]**		
Yes	6,997	7.53
No	85,921	92.47
**Q_3 [c]**		
Yes	9,094	9.79
No	83,820	90.21
**Q_4 [d]**		
Yes	43,651	46.29
No	50,639	53.71
**Number of IT-based Healthcare Communication Strategies**		
0	50,639	53.71
1	32,843	34.83
2	7,020	7.45
3	3,788	4.02

[a] Q_1: Looked-up health information on the internet ("yes" versus "no", and "no" is the reference). [b] Q_2: Scheduled medical appointment on the internet ("yes" versus "no", and "no" is the reference). [c] Q_3: Communicated with healthcare provider by email ("yes" versus "no", and "no" is the reference). [d] Q4: Composite IT-based healthcare communication. This is coded as "Yes" if at least one condition in Q_1- Q_4 were met and coded as "No" otherwise.

2. *Association between Mammography Utilization and IT-based Healthcare Communion- Unadjusted Logistic Regressions*

Based on the results from the univariable logistic regressions, the associations between the IT-based healthcare communication strategies and mammography screening utilization were statistically significant during the entire study period ($p < 0.001$ in all years).

Table S3. Unadjusted logistic regression of mammography utilization based on IT-based healthcare communication. Unadjusted model 0 [a].

Mammography Utilization by time period	Odds Ratio	S.E.	*P*	95% Conf. Interval	
**All years 2011-2018 (n= 94,290)**					
Q_1 [b]	1.66	0.02	<0.001	1.62	1.71
Q_2 [c]	1.87	0.05	<0.001	1.77	1.97
Q_3 [d]	2.00	0.05	<0.001	1.91	2.10
Q_4 [e]	1.72	0.01	<0.001	1.68	1.77
**Year 2011 (n= 11,574)**	1.61	0.06	<0.001	1.49	1.74
Q_1 [b]	2.02	0.22	<0.001	1.64	2.50
Q_2 [c]	1.83	0.16	<0.001	1.53	2.18
Q_3 [d]<br>Q_4 [e]	1.69	0.06	<0.001	1.57	1.82
**Year 2012 (n= 12,306)**	1.65	0.06	<0.001	1.53	1.78
Q_1 [b]	2.26	0.24	<0.001	1.83	2.78
Q_2 [c]	2.11	0.19	<0.001	1.77	2.52
Q_3 [d]<br>Q_4 [e]	1.69	0.06	<0.001	1.57	1.83
**Year 2013 (n= 12,364)**	1.63	0.06	<0.001	1.52	1.76
Q_1 [b]	1.88	0.15	<0.001	1.60	2.21
Q_2 [c]	2.04	0.15	<0.001	1.77	2.36
Q_3 [d]<br>Q_4 [e]	1.66	0.06	<0.001	1.54	1.78
**Year 2014 (n= 13,436)**	1.75	0.06	<0.001	1.63	1.88
Q_1 [b]	2.19	0.18	<0.001	1.86	2.59
Q_2 [c]	2.20	0.16	<0.001	1.91	2.53
Q_3 [d]<br>Q_4 [e]	1.82	0 .06	<0.001	1.70	1.95
**Year 2015 (n= 12,483)**	1.65	0.06	<0.001	1.54	1.77
Q_1 [b]	1.86	0.13	<0.001	1.63	2.13
Q_2 [c]	1.93	0.11	<0.001	1.71	2.17
Q_3 [d]<br>Q_4 [e]	1.74	0.06	<0.001	1.62	1.87
**Year 2016 (n= 12,253)**	1.74	0.06	<0.001	1.62	1.87
Q_1 [b]	1.93	0.12	<0.001	1.70	2.19
Q_2 [c]	2.15	0.12	<0.001	1.91	2.41
Q_3 [d]<br>Q_4 [e]	1.79	0.07	<0.001	1.66	1.92

**Year 2017 (n= 10,099)**	1.60	0.06	<0.001	1.47	1.73
Q_1 [b]	1.58	0.10	<0.001	1.39	1.79
Q_2 [c]	2.00	0.12	<0.001	1.77	2.25
Q_3 [d] Q_4 [e]	1.70	0.07	<0.001	1.57	1.84
**Year 2018 (n= 9,775)**	1.71	0.07	<0.001	1.57	1.86
Q_1 [b]	1.73	0.11	<0.001	1.53	1.96
Q_2 [c]	1.83	0.11	<0.001	1.63	2.06
Q_4 [d] Q_5 [e]	1.84	0.08	<0.001	1.68	2.00

[a] Adjusted model 0: no control variables were included. [b] Q_1: Looked-up health information on the internet ("yes" versus "no", and "no" is the reference). [c] Q_2: Scheduled medical appointment on the internet ("yes" versus "no", and "no" is the reference). [d] Q_3: Communicated with healthcare provider by email ("yes" versus "no", and "no" is the reference). [e] Q_4: Composite IT-based healthcare communication. This is coded as "yes" if at least one condition in Q_1-Q_3 were met and coded as "no" otherwise. "no" is the reference.

3. *The Predicted Probability of Mammography Screening Utilization by the Number of IT-Based Healthcare Communication Strategies*

The analysis showed a statistically significant linear trend in the probability of using mammography screening and the number of IT-based healthcare communication strategies ($P < 0.001$).

Table S4. Linear Trend Analysis in the Probability of Using Mammography Screening based on the Number pf IT Strategies.

	**df** [a]	**Chi²**	***P* > Ch²**
**Number of IT Strategies (Linear)**	1	128.88	<0.001

[a] df: Degree of freedom.

4. *Crosstabulation across different ethnic groups*

Among NH Whites, about 10% were from the lowest income level (<100%) and about 42% were from the highest level (>=400). However, there were about 28% and 29% women in the lowest income level (<100%) and about 18% and 20% from the highest level (>=400) among NH Blacks and Hispanics respectively.

Table S5. Percentages of the ratio of family income to the poverty threshold based on race/ethnicity for women 40+.

	**Poverty ratio level**					
	**<100%**	**100-199%**	**200-399%**	**>=400**	**Total**	***P value*** [a]
**Race/ethnicity**						<0.001
Hispanic	28.86	28.66	24.71	17.77	100	
NH White [b]	9.87	18.39	29.84	41.89	100	
NH Black [c]	27.73	25.61	26.12	20.54	100	
Other	17.63	19.06	24.88	38.44	100	
Total	15.23	20.73	28.38	35.66	100	

[a] *P* value based on Chi2 test. [b] NH White: Non-Hispanic White. [c] NH Black: Non-Hispanic Black.

Around 12 % of NH White women in the sample had less than high school degree and about 30% had a Bachelor' or graduate degree. Among NH Black women, 24% had less than high school degree and 20% had a Bachelor' or graduate degree. Also, around 44% of Hispanic women had less than high school degree and 14% had a Bachelor' or graduate degree.

Table S6. Percentages of the education based on race/ethnicity for women 40+.

	**Education**						
	**Less than high school**	**High school**	**Some college/associate degree**	**Bachelor's degree**	**Graduate degree**	**Total**	***P* value** [a]
**Race/ethnicity**							<0.001
Hispanic	43.80	19.49	22.40	9.30	5.02	100	
NH White [b]	11.90	25.08	31.68	18.72	12.61	100	

NH Black [c]	24.00	24.40	31.54	12.01	8.05	100	
Other	18.45	20.81	22.39	24.88	13.47	100	
Total	17.88	24.05	29.98	16.99	11.10	100	

[a] P value based on Chi2 test. [b] NH White: Non-Hispanic White. [c] NH Black: Non-Hispanic Black.

From the uninsured women in our sample, 30% were Hispanic. In other words, 20.4% of Hispanic women, 10% of NH black women, and 5.6% of NH White women were uninsured. Most of privately insured women in the sample were NH White (75.5%) followed by NH Black (11%), and Hispanic (8%). Insurance disparity could impact the variations in mammography screening utilization.

Table S7. Percentages of the ratio of family income to the poverty threshold based on race/ethnicity for women 40+.

	Insurance coverage						
	**Private**	**Public**	Military	Other	Uninsured	Total	P value [a]
**Race/ethnicity**							<0.001
Hispanic	39.47	35.41	1.81	2.88	20.43	100	
NH White [b]	66.58	22.92	3.90	0.99	5.61	100	
NH Black [c]	46.79	37.96	3.02	2.13	10.10	100	
Other	54.02	30.04	4.58	1.49	9.88	100	
Total	59.73	26.98	3.56	1.41	9.31	100	

[a] P value based on Chi2 test. [b] NH White: Non-Hispanic White. [c] NH Black: Non-Hispanic Black.

Among NH Whites, 4.20 % reported poor health status, 12% reported fair and 21.4 reported excellent. Among NH Blacks, 7.18 reported poor health status, 22.14% reported fair, and 13% reported excellent. Among Hispanics, 6.44% reported poor health status, 22.14% reported fair, 17% reported excellent.

Table S8. Percentages of the health status based on race/ethnicity for women 40+.

	Health status						
	**Poor**	**Fair**	**Good**	**Very good**	**Excellent**	**Total**	***P* value** [a]
**Race/ethnicity**							<0.001
Hispanic	6.44	20.47	32.37	23.85	16.88	100	
NH White [b]	4.20	11.99	28.55	33.89	21.37	100	
NH Black [c]	7.18	22.14	34.36	23.40	12.93	100	
Other	4.55	13.42	34.53	27.46	20.04	100	
Total	4.91	14.54	30.19	30.80	19.55	100	

[a] P value based on Chi2 test. [b] NH White: Non-Hispanic White. [c] NH Black: Non-Hispanic Black.

5. *Association between Mammography Utilization and IT-based Healthcare Communion- Adjusted Logistic Regressions- Model 1*

Table S9 – Table S12 are provided to highlight the significance of education and income in Model 1.

a. *Looking-up health information on the internet (Q_1)*

The results shows that more education and income are associated with higher mammography utilization. In general, with holding Q_1 constant, the odds of mammography utilization among women with bachelor's and graduate's degrees were 1.12 to 1.16 times the utilization of those with less than high school. The odds of mammography utilization among women with family income between 200%-399% of the poverty threshold were 1.14 times the utilization of those making less than 100% the poverty threshold, and the odds were much higher (1.40 times) among those making 400% or more the poverty threshold.

Table S9. Odds ratio and 95% CI based on multiple logistic regression for all years combined (2011-2018) showing all covariates (n= 94,290). Adjusted model 1 [a.]

Mammography Utilization	Odds ratio	S.E.	*P*	95% Conf. Interval	
Q_1 [b] (Baseline: No)					
Yes	1.22	0.02	<0.001	1.18	1.27
Age (Baseline: 40-44)					
45-49	1.66	0.05	<0.001	1.55	1.77
50-54	1.96	0.06	<0.001	1.84	2.08
55-59	2.30	0.07	<0.001	2.17	2.45
65+	2.50	0.06	<0.001	2.64	2.64
Race/ethnicity (Baseline Hispanic)					
NH White [c]	0.69	0.02	<0.001	0.66	0.73
NH Black [d]	0.98	0.03	0.590	0.92	1.05
Other	0.71	0.03	<0.001	0.66	0.77
Marital status (Baseline: Married)					
Widowed	0.69	0.02	<0.001	0.66	0.73
Divorced	0.90	0.02	<0.001	0.86	0.94
Separated	0.86	0.04	0.001	0.79	0.94
Never Married	0.87	0.03	<0.001	0.83	0.92
Living with partner	0.90	0.04	0.021	0.82	0.98
Region (Baseline: East)					
Midwest	1.03	0.02	0.249	0.98	1.08
South	0.90	0.02	<0.001	0.86	0.94
West	0.91	0.02	<0.001	0.86	0.95
Insurance coverage (Baseline: Private)					
Public	0.91	0.02	<0.001	0.87	0.94
Military	1.16	0.05	<0.001	1.07	1.26
Other	1.06	0.07	0.343	0.93	1.21
Uninsured	0.50	0.02	<0.001	0.47	0.54
Education (Baseline: Less than high school)					
High school	1.04	0.03	0.103	0.99	1.09
Some college / Associate degree	1.04	0.03	0.099	0.99	1.10
Bachelor's degree	1.12	0.03	<0.001	1.06	1.019

Graduate degree	1.16	0.04	<0.001	1.08	1.24
Ratio of family income to the poverty threshold (Baseline: <100%)					
100%-199%	0.97	0.03	0.339	0.92	1.03
200%-399%	1.14	0.03	<0.001	1.08	1.21
>=400%	1.40	0.04	<0.001	1.31	1.48
Work status (Baseline: No)					
Yes	1.07	0.02	<0.001	1.03	1.11
Place usually go when sick (Baseline: No)					
Yes	2.34	0.09	<0.001	2.16	2.53
Physical health status (Baseline: Poor)					
fair	1.16	0.05	<0.001	1.07	1.26
Good	1.40	0.05	<0.001	1.30	1.51
Very good	1.56	0.06	<0.001	1.45	1.69
Excellent	1.59	0.06	<0.001	1.47	1.72
Seen/talked to a general doctor (Baseline: No)					
Yes	1.98	0.04	<0.001	1.90	2.06
Seen/talked to OB/GYN					
Yes	3.50	0.06	<0.001	3.37	3.63
Year (Baseline: 2011)					
2012	1.12	0.03	<0.001	1.05	1.19
2013	0.76	0.02	<0.001	0.71	0.81
2014	0.97	0.03	0.263	0.91	1.02
2015	0.69	0.02	<0.001	0.65	0.74
2016	0.98	0.03	0.563	0.92	1.04
2017	0.94	0.03	<0.001	0.88	1.00
2018	0.88	0.03	<0.001	0.82	0.94

[a] Adjusted model 1: adjusting for age, race/ethnicity, marital status, education, region, insurance coverage, work status, place usually go when sick, seen/talked to a general doctor, seen/talked to OB/GYN, ratio of family income to the poverty threshold, and physical health status. [b] Q_1: Looked-up health information on the internet ("yes" versus "no", and "no" is the reference). [c] NH White: Non-Hispanic White. [d] NH Black: Non-Hispanic Black.

b. Scheduling medical appointments on the internet (Q2)

With holding Q_2 constant, the increase in the odds of mammography among women with high school, associate, bachelor's and graduate's degrees ranged from 6% to 23% compared to those with less than high school. The odds of mammography utilization among women with family income between 200%-399% of the poverty threshold were 1.16 times the utilization of those making less than 100% the poverty threshold, and the odds were much higher (1.43 times) among those making 400% or more the poverty threshold.

Table S10. Odds ratio and 95% CI based on multiple logistic regression for all years combined (2011-2018) showing all covariates (n= 94,290). Adjusted model 1 [a].

Mammography Utilization	Odds ratio	S.E.	*P*	95% Conf. Interval	
Q_2 [b] (Baseline: No)					
Yes	1.23	0.04	<0.001	1.16	1.30
Age (Baseline: 40-44)					
45-49	1.66	0.05	<0.001	1.55	1.77
50-54	1.95	0.06	<0.001	1.83	2.08
55-59	2.30	0.07	<0.001	2.16	2.44
65+	2.45	0.07	<0.001	2.32	2.59
Race/ethnicity (Baseline Hispanic)					
NH White [c]	0.72	0.02	<0.001	0.068	0.75
NH Black [d]	0.99	0.03	0.629	0.93	1.05
Other	0.71	0.03	<0.001	0.66	0.77
Marital status (Baseline: Married)					
Widowed	0.68	0.01	<0.001	0.65	0.71
Divorced	0.90	0.02	<0.001	0.87	0.94
Separated	0.86	0.03	0.001	0.78	0.94
Never Married	0.87	0.02	<0.001	0.82	0.92
Living with partner	0.90	0.04	0.024	0.82	0.99
Region (Baseline: East)					
Midwest	1.03	0.02	0.225	0.98	1.08
South	0.90	0.02	<0.001	0.86	0.94
West	0.90	0.02	<0.001	0.86	0.95
Insurance coverage (Baseline: Private)					
Public	0.91	0.02	<0.001	0.87	0.95
Military	1.16	0.05	<0.001	1.07	1.26
Other	1.07	0.07	0.319	0.94	1.22
Uninsured	0.51	0.02	<0.001	0.47	0.54
Education (Baseline: Less than high school)					
High school	1.06	0.03	0.020	1.01	1.11
Some college / Associate degree	1.09	0.03	0.001	1.04	1.14
Bachelor's degree	1.19	0.03	<0.001	1.12	1.26
Graduate degree	1.23	0.04	<0.001	1.15	1.31
Ratio of family income to the poverty threshold (Baseline: <100%)					
100%-199%	0.98	0.02	0.438	0.93	1.03
200%-399%	1.16	0.03	<0.001	1.10	1.23
>=400%	1.43	0.04	<0.001	1.34	1.52
Work status (Baseline: No)					
Yes	1.08	0.02	<0.001	1.04	1.13
Place usually go when sick (Baseline: No)					
Yes	2.33	0.09	<0.001	2.16	2.52
Physical health status (Baseline: Poor)					
fair	1.17	0.05	<0.001	1.08	1.26
Good	1.40	0.05	<0.001	1.30	1.51
Very good	1.57	0.06	<0.001	1.45	1.70

Excellent	1.59	0.06	<0.001	1.47	1.72
Seen/talked to a general doctor (Baseline: No)					
Yes	1.99	0.04	<0.001	1.91	2.07
Seen/talked to OB/GYN					
Yes	3.52	0.06	<0.001	3.40	3.66
Year (Baseline: 2011)					
2012	1.11	0.03	0.001	1.05	1.18
2013	0.76	0.02	<0.001	0.72	0.81
2014	0.96	0.03	0.188	0.91	1.02
2015	0.69	0.02	<0.001	0.65	0.74
2016	0.98	0.03	0.541	0.92	1.04
2017	0.94	0.03	0.071	0.89	1.00
2018	0.88	0.03	<0.001	0.82	0.94

[a] Adjusted model 1: adjusting for age, race/ethnicity, marital status, education, region, insurance coverage, work status, place usually go when sick, seen/talked to a general doctor, seen/talked to OB/GYN, ratio of family income to the poverty threshold, and physical health status. [b] Q_2: Scheduled medical appointment on the internet ("yes" versus "no", and "no" is the reference). [c] NH White: Non-Hispanic White. [d] NH Black: Non-Hispanic Black.

c. Communicated with healthcare provider using Email (Q_3)

With holding Q_3 constant, the increase in the odds of mammography among women with high school, associate, bachelor's and graduate's degrees were 6%, 8%, 18% and 20% compared to those with less than high school. The odds of mammography utilization among women with family income between 200%-399% of the poverty threshold were 1.16 times the utilization of those making less than 100% the poverty threshold, and the odds were much higher (1.42 times) among those making 400% or more the poverty threshold.

Table S11. Odds ratio and 95% CI based on multiple logistic regression for all years combined (2011-2018) showing all covariates (n= 94,290). Adjusted model 1 [a].

Mammography Utilization	Odds ratio	S.E.	*P*	95% Conf. Interval	
Q_3 [b] (Baseline: No)					
Yes	1.33	0.03	<0.001	1.26	1.40
Age (Baseline: 40-44)					
45-49	1.66	0.06	<0.001	1.55	1.77
50-54	1.96	0.06	<0.001	1.84	2.08
55-59	2.29	0.07	<0.001	2.15	2.44
65+	2.45	0.07	<0.001	2.32	2.59
Race/ethnicity (Baseline Hispanic)					
NH White [c]	0.71	0.02	<0.001	0.67	0.75
NH Black [d]	0.98	0.03	0.640	0.92	1.05
Other	0.71	0.03	<0.001	0.66	0.77
Marital status (Baseline: Married)					
Widowed	0.68	0.01	<0.001	0.65	0.71
Divorced	0.90	0.02	<0.001	0.86	0.94

Separated	0.86	0.04	0.001	0.78	0.94
Never Married	0.86	0.02	<0.001	0.82	0.91
Living with partner	0.90	0.04	0.022	0.82	0.98
Region (Baseline: East)					
Midwest	1.03	0.02	0.265	0.98	1.08
South	0.90	0.02	<0.001	0.86	0.94
West	0.90	0.02	<0.001	0.85	0.94
Insurance coverage (Baseline: Private)					
Public	0.91	0.02	<0.001	0.87	0.95
Military	1.16	0.05	<0.001	1.07	1.26
Other	1.07	0.07	0.326	0.94	1.21
Uninsured	0.51	0.02	<0.001	0.47	0.54
Education (Baseline: Less than high school)					
High school	1.06	0.03	0.021	1.01	1.11
Some college / Associate degree	1.08	0.03	0.001	1.03	1.14
Bachelor's degree	1.18	0.03	<0.001	1.11	1.25
Graduate degree	1.20	0.04	<0.001	1.13	1.29
Ratio of family income to the poverty threshold (Baseline: <100%)					
100%-199%	0.98	0.03	0.430	0.93	1.03
200%-399%	1.16	0.03	<0.001	1.10	1.22
>=400%	1.42	0.04	<0.001	1.33	1.51
Work status (Baseline: No)					
Yes	1.08	0.02	<0.001	1.04	1.12
Place usually go when sick (Baseline: No)					
Yes	2.32	0.09	<0.001	2.15	2.51
Physical health status (Baseline: Poor)					
fair	1.16	0.05	<0.001	1.08	1.26
Good	1.40	0.05	<0.001	1.30	1.51
Very good	1.57	0.06	<0.001	1.45	1.70
Excellent	1.59	0.06	<0.001	1.47	1.73
Seen/talked to a general doctor (Baseline: No)					
Yes	1.98	0.04	<0.001	1.90	2.06
Seen/talked to OB/GYN					
Yes	3.52	0.07	<0.001	3.39	3.65
Year (Baseline: 2011)					
2012	1.11	0.03	0.001	1.05	1.18
2013	0.76	0.02	<0.001	0.71	0.81
2014	0.96	0.03	0.152	0.90	1.01
2015	0.69	0.02	<0.001	0.65	0.73
2016	0.97	0.03	0.404	0.92	1.03
2017	0.93	0.03	0.038	0.88	0.10
2018	0.87	0.03	<0.001	0.81	0.93

[a] Adjusted model 1: adjusting for age, race/ethnicity, marital status, education, region, insurance coverage, work status, place usually go when sick, seen/talked to a general doctor, seen/talked to OB/GYN, ratio of family income to the poverty threshold, and physical health status. [b] Q_3: Communicated with healthcare provider by email (“yes”

versus "no", and "no" is the reference). [c] NH White: Non-Hispanic White. [d] NH Black: Non-Hispanic Black.

d. Composite IT-based healthcare communication (Q_4)

With holding Q_4 constant, the odds of mammography utilization among women with bachelor's and graduate's degrees were 1.11 to 1.14 times the utilization of those with less than high school. The odds of mammography utilization among women with family income between 200%-399% of the poverty threshold were 1.14 times the utilization of those making less than 100% the poverty threshold, and the odds were much higher (1.39 times) among those making 400% or more the poverty threshold. We are making more explicit in the manuscript that our results hold after controlling for education and income, and we now also highlight the statistically significant association of those two variables with mammography utilization.

Table S12. Odds ratio and 95% CI based on multiple logistic regression for all years combined (2011-2018) showing all covariates (n= 94,290). Adjusted model 1 [a].

**Mammography Utilization**	**Odds ratio**	**S.E.**	***P***	**95% Conf. Interval**	
Q_4 [b] (Baseline: No)					
Yes	1.27	0.02	<0.001	1.23	1.32
Age (Baseline: 40-44)					
45-49	1.66	0.06	<0.001	1.55	1.77
50-54	1.96	0.06	<0.001	1.85	2.09
55-59	2.31	0.07	<0.001	2.17	2.46
65+	2.52	0.07	<0.001	2.39	2.67
Race/ethnicity (Baseline Hispanic)					
NH White [c]	0.69	0.02	<0.001	0.65	0.73
NH Black [d]	0.98	0.03	0.592	0.92	1.05
Other	0.72	0.03	<0.001	0.66	0.77
Marital status (Baseline: Married)					
Widowed	0.70	0.02	<0.001	0.67	0.73
Divorced	0.90	0.02	<0.001	0.86	0.94
Separated	0.86	0.04	0.001	0.78	0.94
Never Married	0.87	0.02	<0.001	0.83	0.92
Living with partner	0.90	0.04	0.021	0.82	0.98
Region (Baseline: East)					
Midwest	1.03	0.02	0.267	0.98	1.08
South	0.90	0.02	<0.001	0.86	0.94
West	0.90	0.02	<0.001	0.86	0.95
Insurance coverage (Baseline: Private)					
Public	0.91	0.02	<0.001	0.87	0.95
Military	1.16	0.05	<0.001	1.07	1.26
Other	1.07	0.07	0.328	0.94	1.21
Uninsured	0.50	0.02	<0.001	0.47	0.54
Education (Baseline: Less than high school)					
High school	1.04	0.03	0.139	0.99	1.09

Some college / Associate degree	1.03	0.03	0.251	0.98	1.08
Bachelor's degree	1.11	0.03	0.001	1.04	1.18
Graduate degree	1.14	0.04	<0.001	1.06	1.22
Ratio of family income to the poverty threshold (Baseline: <100%)					
100%-199%	0.97	0.03	0.323	0.92	1.03
200%-399%	1.14	0.03	<0.001	1.07	1.20
>=400%	1.38	0.04	<0.001	1.30	1.47
Work status (Baseline: No)					
Yes	1.07	0.02	0.001	1.03	1.11
Place usually go when sick (Baseline: No)					
Yes	2.33	0.09	<0.001	2.16	2.52
Physical health status (Baseline: Poor)					
fair	1.16	0.05	<0.001	1.08	1.26
Good	1.40	0.05	<0.001	1.30	1.51
Very good	1.56	0.06	<0.001	1.45	1.69
Excellent	1.59	0.07	<0.001	1.47	1.72
Seen/talked to a general doctor (Baseline: No)					
Yes	1.97	0.04	<0.001	1.89	2.05
Seen/talked to OB/GYN					
Yes	3.49	0.06	<0.001	3.36	3.62
Year (Baseline: 2011)					
2012	1.11	0.03	<0.001	1.05	1.18
2013	0.76	0.02	<0.001	0.71	0.80
2014	0.96	0.03	0.223	0.91	1.02
2015	0.69	0.02	<0.001	0.64	0.73
2016	0.97	0.03	0.417	0.91	1.03
2017	0.93	0.03	0.033	0.88	0.99
2018	0.87	0.03	<0.001	0.81	0.93

[a] Adjusted model 1: adjusting for age, race/ethnicity, marital status, education, region, insurance coverage, work status, place usually go when sick, seen/talked to a general doctor, seen/talked to OB/GYN, ratio of family income to the poverty threshold, and physical health status. [b] Q_4: Composite IT-based healthcare communication. This is coded as "yes" if at least one condition in Q1-Q4 were met and coded as "no" otherwise. "no" is the reference. [c] NH White: Non-Hispanic White. [d] NH Black: Non-Hispanic Black.

6. Directed Acyclic Graphs

The confounding variable must influence both the exposure and the outcome.

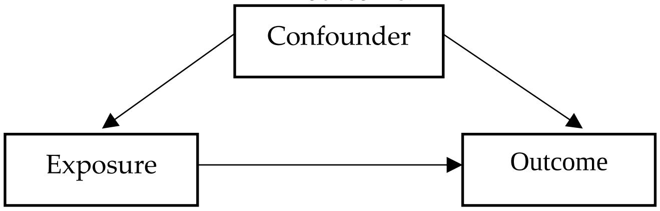

Figure S1. Confounding Variable

The mediator variable explains the relationship between the dependent variable and the independent variable

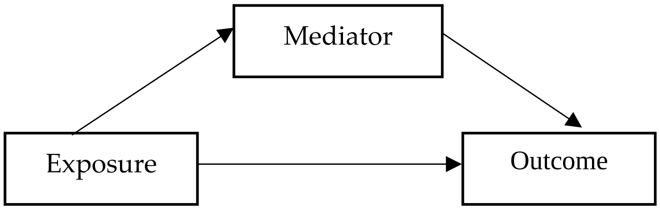

Figure S2. Mediator Variable
